# Nurses’ Knowledge and Attitudes towards Prevention of Pressure Ulcers

**DOI:** 10.3390/ijerph18041705

**Published:** 2021-02-10

**Authors:** Beáta Grešš Halász, Anna Bérešová, Ľubomíra Tkáčová, Dagmar Magurová, Ľubomíra Lizáková

**Affiliations:** 1 Department of Nursing, Faculty of Health Care, University of Prešov, Partizánska 1, 080 01 Prešov, Slovakia; lubomira.tkacova@unipo.sk (Ľ.T.); dagmar.magurova@unipo.sk (D.M.); lubomira.lizakova@unipo.sk (Ľ.L.); 2 Department of Social and Behavioral Medicine, Faculty of Medicine, University of P. J. Šafárik, Tr. SNP 1, 040 01 Košice, Slovakia; anna.beresova@upjs.sk

**Keywords:** knowledge, attitude, pressure ulcer, prevention, nurses

## Abstract

Background: Pressure ulcers (PU) remain a serious complication of immobile patients and a burden for healthcare professionals. The incidence and prevalence remain alarming. Knowledge and attitudes of nurses play a fundamental role in prevention. The aim of this study was to determine the knowledge and attitudes of nurses towards the prevention of PU in selected Slovak hospitals and find relationships and differences among selected variables. Methods: A quantitative exploratory cross-sectional design was chosen. Validated instruments were used. From the 460 randomly selected nurses, 225 (49%) participated in this research. Results: Results showed insufficient knowledge (45.5%) and attitudes (67.9%) of nurses towards PU prevention. There was a significant positive correlation found between the knowledge and attitudes (ρ = 0.300; *p* = 0.000). Nurses´ knowledge was significantly different within the level of education (*p* = 0.031) and work department (*p* = 0.048). Conclusions: Results showed insufficiencies in the knowledge and attitudes of nurses towards PU prevention. Therefore, it is essential to focus on general education and continuing education and practice of nurses. Further development of educational programs and frequent measurement of these two parameters can lead to a significant improvement in the quality of care provided.

## 1. Introduction

Pressure ulcers are painful burden for patients/clients of all ages, which causes complications as comfort, pain, quality of life, costs and a long stay in hospitals. They might result in a life-threatening situation. The issue of pressure ulcer incidence is very complex. It includes regulations and auditing, implementation of adequate preventive and treatment procedures, resources, evidence-based practice, educated staff and active involvement of professionals. Despite progressive technologies and successful clinical researches in terms of prevention and treatment, pressure ulcers present high incidence of 7–71.6% [1,2,3,4], prevalence of 8.8–53.2% [5,6,7], and considerably high mortality [8]. The cost of pressure ulcer prevention varies between 2.65€ to 87.57€ a day per patient while the cost of pressure ulcer treatment ranged between 1.71€ to 470.49€ a day per patient [9]. Monitoring the incidence of pressure ulcers in Slovakia has not been unified yet; the problem rests in the inconsistency of evaluation and standardization of pressure ulcers prevention and treatment, insufficiencies in reviews and audits, missing methodological guidance, preventive programs and relevant data collection [10]. Reviewing accessible information, National Centre of Health Information (NCZI) and insurance companies provided their databases of reported pressure ulcers by health care facilities. The final calculation carried out by authors of this research resulted in a very low-pressure ulcer incidence of 0.05%. It must be mentioned that information provided by NCZI and insurance companies slightly differ. For all these reasons, it could be argued whether reported numbers of pressure ulcers by Slovak health care facilities are veracious.

Regulation and auditing of pressure ulcers incidence are included in processes and procedures of quality control led and regulated by the Ministry of Health of Slovak Republic (MZ SR). Health care facilities document and report numbers of pressure ulcers (with unspecified stage) to legal bodies on regular bases. Monitoring of incidence and prevalence of pressure ulcers in Slovakia is not sufficiently recorded, controlled, audited, reviewed and publicized [10]. These facts confirm that the management of pressure ulcers might be a grey area of the Slovak health care system.

Adequate standards of care related to pressure ulcers should be implemented on all levels of care and should be one of the priorities of any hospital and home care setting to deliver adequate and high quality of care [11,12,13]. Standardized instructions can significantly prevent pressure ulcers [14,15]. European Pressure Ulcer Advisory Panel (EPUAP) sets and regularly reviews standards and procedures of pressure ulcer management on bases of research. In many cases, it was found that standards and procedures are not used or used insufficiently [16,17]. Standards for pressure ulcer care for the Slovak health care system were formulated more than 10 years ago [18]. They have not been updated since, and it can be seen as a deficiency [10].

Knowledge represents the ground for practice leading to quality and safety of care provided. Most research worldwide shows a deficiency in knowledge and inadequate attitudes of nurses in this field of interest. This phenomenon has not changed much in the past 15–20 years. Several studies used the Pieper´s Pressure Ulcer Knowledge Test (PUKT) to examine nurses´ knowledge in pressure ulcer management. Older researches tested pressure ulcer knowledge of registered nurses. The more recently had participants read about pressure ulcers, the higher scores they reached. There was no relation between the knowledge and age, educational background, or years of nursing experience found [19]. Critical care nurses´ knowledge of pressure ulcer prediction and prevention revealed a knowledge deficit in the area of interest among critical care nurses. No affection of years of nursing experience, type of nursing education, or recent interest in reading articles about pressure ulcer management on the knowledge was found. Only a few nurses had read guidelines on pressure ulcer prevention [20]. Very similar results were revealed in other studies. Undergraduate student nurses scored low. Respondents participating in extracurricular activities scored better [21,22]. Nurses working in medical and nephrology wards, nurses who undertook pressure ulcer training, and nurses who perceived their knowledge to be better scored significantly higher. Wide discrepancies were found between risk factors and preventive interventions [23].

Aydin and Karadag assessed nurses´ knowledge related to deep tissue injury and pressure ulcer stage 1. They constructed and evaluated the used questionnaire. The majority of respondents were found to have a significant lack of knowledge in the assessment and management of deep tissue injury and pressure ulcer stage 1. Respondents with baccalaureate and master´s degree as well as those with pressure ulcer educational program background scored better [24]. Pressure ulcer knowledge of preventive methods and management was below the acceptable level [25,26].

Reviewing the literature, only a few researches resulted in a satisfactory level of knowledge. Pancorbo- Hidalgo et al. using a self-constructed and validated questionnaire looked at respondents´ pressure ulcer knowledge of guidelines, its implementation, and influencing factors. Respondents showed good knowledge but insufficient implementation of the guidelines. The level of education, pressure ulcer training, and years of professional experience had a significant influence on results [17]. The aim of the study of Källman and Suserud was to review the level of knowledge, attitudes, the practice of assessment, and perceived barriers to pressure ulcer prevention. Using an adapted measurement instrument of authors Moore and Price and Lewin et al., nurses and nursing assistants demonstrated good knowledge and positive attitudes towards pressure ulcer prevention and treatment [27]. Moreover, Strand and Lindgren in their study questioned nurses working in an intensive care unit using the same questionnaire as in the previous study by Källman and Suserud, where nurses´ knowledge and attitudes were found to be satisfactory. However, significant score differences between registered nurses and enrolled nurses were revealed [28]. Barriers to carry out satisfactory preventive methods were lack of time and patients´ condition [22,27,28], knowledge, and access to preventive equipment [28], and lack of equipment or resources [27]. Professor Beeckman et al. tested the knowledge and attitude scores using PUKAT (knowledge) and APuP (attitudes) tools. Both results testing Belgian nurses were low. Furthermore, the application of adequate prevention in a nursing ward was significantly correlated with the attitudes of the nurses. No correlation was found between knowledge and the application of adequate prevention [26]. We conclude that knowledge and attitudes towards pressure ulcers´ prevention play a fundamental role in practice.

As the literature review has shown, knowledge and attitudes of nurses towards pressure ulcer management are mostly deficient. Differences among nurses with or without pressure ulcer training or with a university degree or tissue viability education are significant. Barriers to implement adequate preventive methods to patients/clients at risk of developing a pressure ulcer are similar in most studies. Our research review issues related to nurses´ knowledge and attitudes towards management of pressure ulcers offers an insight to a condition in four major Eastern Slovak hospitals and compares the results with other countries. Slovak hospitals are lacking research regarding the knowledge and attitudes towards pressure ulcer management. The authors, therefore, decided to carry out research related to this topic using a current tool in the hope that it will be able to create a basis for further research in Slovak health care facilities.

## 2. Materials and Methods

A quantitative exploratory cross-sectional design was chosen to conduct this study. Participants were introduced to a knowledge test and attitude assessment, from which the ethics committees and management of each hospital where the study was conducted confirmed their use.

Six major East Slovakian hospitals in two large cities, Košice and Prešov, were selected, of which four hospitals positively replied to our request, but a long-term geriatric setting did not wish to take part, and a major geriatric hospital did not answer at all. We personally distributed instruments to each nurse manager and collected them from January to March 2017. Inclusion and exclusion criteria for participation was specified on the bases of the aim of this study. Nurses providing direct nursing care to patients in acute or long-term wards willing to participate in the research were included. The study population of 460 randomly selected nurses was invited to take part. The final response rate was 49% (225 nurses).

Measurement instruments PUKAT (Pressure Ulcer Knowledge Assessment Tool) [29] and APuP (Attitudes towards Pressure Ulcers Prevention tool) [30] were adapted, translated, retranslated from the original English version to Slovak language, and the final validated instruments were used for this research work. Both instruments were constructed and rigorously tested by Beeckman et al. in 2010. Tools are considered to be brief and conceptually sound with the evidence of supporting the psychometric properties [25]. PUKAT instrument was extensively validated in terms of items difficulty, discriminating index, and the quality of response alternatives. The internal consistency reliability (Cronbach α) was 0.77, and the 1- week test–retest interclass correlation coefficient (stability) was 0.88. In this study, Cronbach α was tested and its result index was 0.514 [29].

PUKAT assessment instrument used for testing participants´ knowledge of prevention of pressure ulcers included 26 questions grouped in 6 domains of pressure ulcer prevention: (1) etiology and development, (2) classification and observation, (3) nutrition, (4) risk assessment, (5) reduction of the magnitude of pressure and tearing, and (6) reduction of the duration of pressure and shearing. Respondents could choose from several answers, where only one was correct. The maximum score of correct responses was 26, and the mean of ≥60% was considered to be satisfactory [29].

The Content Validity Index of the constructed APuP tool by Beeckman et al. was between 0.87 and 1.00 and Cronbach´s α ranged from 0.76 to 0.81. Testing Cronbach α of the APuP tool in this study resulted in 0.938. Attitudes of respondents toward pressure ulcers management were assessed by APuP measurement tool. The 13-item instrument was divided into 5 domains: (1) personal competency to prevent pressure ulcers, (2) impact of pressure ulcer prevention, (3) impact of pressure ulcers, (4) responsibility of pressure ulcer prevention, and (5) confidence in the effectiveness of prevention. Opinions were possible to express on a Likert-type scale (1 = strongly agree, 2 = agree, 3 = disagree, 4 = strongly disagree). Negatively worded statements had reversed coding. The total score that could be obtained was 52, and the satisfactory mean represented ≥75% [30].

The final instrument comprised three main parts: demographics, PUKAT, and APuP. Respondents completed instruments anonymously.

Collected data were statistically analyzed using SPSS 20. Initial data were expressed in descriptive frequencies and statistical means. A correlation test was performed to determine relationships among variables by Spearman ρ value to give a notion of the strength of correlation and a significance expressed in *p* value < 0.05. ANOVA was carried out to test differences among scores and score groups.

The Ethical committees of four hospitals approved this study. The permission to use PUKAT and APuP instruments was provided by the author, prof. Beeckman from the University of Gent, Belgium. The study population was randomly selected on the bases of inclusion and exclusion criteria, and it was presented with an invitation letter attached to the final instrument. Research packs were distributed to nurse managers of each hospital who further presented these to the nurses. The study population was informed about the purpose, procedure, compliance with confidentiality and anonymity within the study, and was asked to complete the instrument individually without the use of other sources. The completed instrument was an expression of consent to participate. Participants had a choice of completion of the instrument on paper or online. The approximate time of completion was 20 min.

## 3. Results

### 3.1. Demographics

Six major Eastern Slovak hospitals in Košice and Prešov were addressed to participate in the descriptive research that aimed to explore the knowledge and attitudes of nurses towards pressure ulcers´ prevention. Four hospitals agreed and issued an ethical approval (one hospital in Prešov and three hospitals in Košice). The response rate was 49.10% (*n* = 225) of which 66.20% (*n* = 149) of respondents were from hospitals in Košice and 33.80% (*n* = 76) from the hospital in Prešov. The mean age of all respondents was 39.47 years (SD ± 10.10), and the mean of years of nursing experience was 17.91 years (SD ± 11.28). From the total, 88.00% were staff nurses and 9.00% nurse managers. The majority of respondents worked in surgical (24.4%) and medical (14.2%) wards, neurology (12.9%), oncology (12.9%), intensive care units (11.6%), and trauma wards (10.7%). Most of the nurses completed a specialty education in their field (25.3%) and first level university degree (BSc.) (23.1%). Slightly fewer nurses completed a secondary nursing school (17.3%), higher diploma in nursing (16.00%), second level of university degree (MSc.) (16.0%), and at least Doctor in Nursing (DN) (1.8%). Only 1.3% of respondents undertook a course for pressure ulcer management. The majority of 73.8% reported pressure ulcer education completed within the basic nursing education and 20.4% within continual nursing education. The rest of the respondents reported that the information related to pressure ulcer prevention had been acquired through self-study, via the internet, or in practice. Demographic data are listed in Table 5 in details.

### 3.2. Knowledge and Attitudes

Analyses of data showed the mean score of knowledge 45.5% (M = 11.84; SD ± 2.83). Only 9.0% (*n* = 21) of nurses succeeded in reaching ≥60%, which is considered to be the limit by authors of PUKAT instrument. The most successful group domain was nutrition (77.0%), and the least successful was risk assessment (38.5%) (Table 1).

Attitudes were more positive. However, the mean score was only 67.9% (M = 35.28; SD ± 11.99) which again did not meet the satisfactory limit of ≥75% set by the authors of APuP tool. Only 56.4% (*n* = 127) of respondents reached this limit. All five-domain group mean scores were between 67–69% (Table 2).

### 3.3. Correlations and Differences

Correlations among variables were tested by Spearman ρ. Medium positive correlation was found between the knowledge and attitudes of respondents (ρ = 0.300; *p* = 0.000). The most significant correlated domains were etiology and development (knowledge) and responsibility in pressure ulcer prevention (attitudes) (ρ = 0.252; *p* = 0.000); risk assessment (knowledge) and responsibility in pressure ulcer prevention (attitudes) (ρ = 0.263; *p* = 0.000) (Table 3). No other significant correlations were found.

Differences among variables were tested using ANOVA test formats. Statistically significant differences were found between the mean knowledge of nurses working on the trauma wards (49.35%) and neurology wards (39.65%) (F = 2.07; *p* = 0.048) and the mean knowledge of nurses with secondary nursing education (49.73%) and first level university degree (42.45%) (F = 2.51; *p* = 0 031) (Table 5). Correlations between age or years of practice and knowledge or attitudes were tested; however, no significances were found (Table 4). Moreover, no further significant differences were found among all tested variables (Table 5).

## 4. Discussion

This descriptive study aimed to define the knowledge and attitudes towards pressure ulcer prevention of nurses from four major Eastern Slovak hospitals. Five hypotheses were set to test the level of the knowledge, the attitudes, correlations and differences among variables. The results showed a lack of knowledge and attitudes to prevent pressure ulcers. This suggests that there are no major differences between most countries.

Reviewing the literature, the majority of researches mostly show lack of knowledge [16,19,20,21,22,23,24,25,26,31,32] and attitudes [16,26] towards the management of pressure ulcers among nurses as same as it was revealed in this research. Few results showed satisfactory pressure ulcer knowledge [17,27,28,31,33] and attitudes [27,31,32,33] among nursing staff. In our study, knowledge and attitudes correlated positively and were statistically significant. The same result was found in other studies [31,32]. Nurses with higher education scored better in most studies [17,24,26], although a few older works showed no significances in education [19,20] or years of nursing experience [19,20,21,22]. This study showed that nurses with a bachelor’s degree scored less than nurses with secondary nursing education due to the changed system and content of nursing education in Slovakia in the late nineties. Reading articles by nurses about pressure ulcers´ prevention has no significant effect on their knowledge [20]. However, educational programs and self-study have the potential to positively impact nurses’ knowledge related to pressure ulcers´ prevention in most of the studies [17,19,21,22,23,24,26]. Positive influence in years of nursing experience on pressure ulcer knowledge was revealed in two studies [17,27]. Years of experience have no significant influence on the reached knowledge scores of respondents in this research. In some studies, serious issues were revealed: patients not receiving adequate prevention [16,26], insufficiencies in compliance with preventive methods and guidelines [16,17], and wide discrepancies between risk factors and preventive interventions [23]. Compliance with preventive methods and guidelines were not reviewed in this research, therefore it could not be tested and compared with the literature.

Comparing our results with the study of Beeckman et al. [26], which was the model for this work, Belgian nurses scored better than Slovak nurses in both knowledge and attitudes. In the knowledge test, only 9% of all respondents reached the level of ≥60%, which was significantly less than Belgian nurses, who reached the limit by 24%. Assessing attitudes, only 56% of Slovak nurses reached the limit of ≥75%, but overall, they scored better than Belgian nurses (51%). Belgian nurses who received further training in pressure ulcer prevention scored higher than those who did not. This aspect was irrelevant for comparison, as only 1% of Slovak nurses stated that they had completed a pressure ulcer management course. Nursing education was one of the factors that showed significances in differences within the knowledge of Slovak nurses as well as of Belgian nurses. However, the interesting finding was that Slovak nurses who completed a secondary nursing school scored significantly higher than nurses with a bachelor’s degree as opposed to Belgian nurses where nurses with bachelor’s degree scored significantly higher than certified nurses. Additionally, there were significant differences in knowledge found between nurses working on trauma wards in comparison to nurses working on neurology wards. We presume, that nurses working on trauma wards may be more advanced in the treatment of wounds and therefore may be more inclined to the prevention of pressure ulcers.

Pressure ulcers remain a serious issue worldwide. Despite advanced technologies and rapid development of preventive methods, pressure ulcers continue to be a cause of severe health complication and high mortality. Incidence of pressure ulcers is an important indicator of quality care provided by professionals. It seems that it is not sufficiently controlled in the Slovak health care system. According to the available information, the incidence of pressure ulcers is scant. Data collection and reporting is a responsibility of settings who may try not to compromise the quality of provided care. Moreover, the results of this research show a deficient knowledge and attitudes towards pressure ulcers´ prevention. Therefore, it is questionable whether the incidence is sufficiently recorded and the data show the reality. The aim should focus on areas of incidence of pressure ulcers, the use of preventive guidelines and their relevance, the knowledge and attitudes of health care professionals related to pressure ulcers, and the education and its system in tissue viability and wound care nationwide.

## 5. Conclusions

In comparison to other countries, according to available information, incidence levels of pressure ulcers in Slovakia appear very low. However, monitoring and control of the occurrence of pressure ulcers in Slovak health care facilities is debatable. The aim of the authors was to determine the level of knowledge and attitudes to the prevention of pressure ulcers as a preliminary to a complex care management of pressure ulcers and to compare results with other studies to draw implications for future research related to education and practice. The set aims of the study were completed. Results revealed unsatisfactory knowledge (45.5%) and low attitudes (67.9%) of nurses towards prevention of pressure ulcers, positive correlation between them (ρ = 0.300; *p* = 0.000), and significant difference of knowledge between levels of education (*p* = 0.031) and work departments (*p* = 0.048). Addressing the findings of the research could provide a clearer view of the current content of education and practice of nurses on pressure ulcers and can create a basis for pedagogical and practical training and motivation of health professionals in wound management and tissue viability in Slovakia in order to increase quality of provided care.

The purpose of this study was to determine the knowledge and attitudes of nurses towards the prevention of PU in selected Slovak hospitals as a prerequisite of pressure ulcer care and management and find relationships and differences among selected variables. Based on our findings, several consequences have emerged:

- Results of the research showed insufficient knowledge and attitudes of nurses towards PU prevention in Eastern Slovak hospitals. Due to the specific outcomes, we suggest reviewing and comparing the curricula of the nursing education at graduate, postgraduate, and specialty levels. It would be highly appropriate to review the continuing education and practice of nurses too.
- No research work related to pressure ulcer prevention or management carried out in Slovak hospitals was found. We suggest conducting an in- depth research at the national level, focusing on resulting topics.

## Figures and Tables

**Table 1:** Descriptive statistics of Knowledge (domains and total).

Domain (1.–6.)	%	M/SD±	Max. Score
1. Etiology and development	39.50	2.37/1.13	6
2. Classification and observation	48.20	2.41/0.99	5
3. Nutrition	77.00	0.77/0.65	1
4. Risk assessment	38.50	0.77/0.42	2
5. Eeduction of the magnitude of pressure and tearing	43.00	3.01/1.28	7
6. Reduction of the duration of pressure and shearing	51.20	2.56/1.06	5
Total Knowledge	45.50	11.84/2.83	26

**Table 2:** Descriptive statistics of Attitudes (domains and total).

Domain (1.–5.)	%	M/SD±	Max. Score
1. Personal competency to prevent pressure ulcers	68.75	8.25/3.00	12
2. Impact of pressure ulcer prevention	67.08	8.05/3.04	12
3. Impact of pressure ulcers	67.30	8.08/3.29	12
4. Responsibility of pressure ulcer prevention	68.88	5.51/1.95	8
5. Confidence in the effectiveness of prevention	68.00	5.39/1.83	8
Total Attitudes	67.90	35.28/11.99	52

**Table 3:** Significant correlations among variables.

Correlated Variables	Correlations
Knowledge/Attitude	(ρ = 0.300; *p* = 0.000 ***)
1. Etiology and development (knowledge)/ 4. Responsibility in pressure ulcer prevention (attitudes)	(ρ = 0.252; *p* = 0.000 ***)
4. Risk assessment (knowledge)/ 4. Responsibility of pressure ulcer prevention (attitudes)	(ρ = 0.263; *p* = 0.000 ***)

Notes: *** *p*< 0.001.

**Table 4:** Correlations between variables.

Correlated Variables	Correlations
Knowledge/Age	(ρ = 0.125; *p* = 0.062)
Attitudes/Age	(ρ = −0.089; *p* = 0.183)
Knowledge/Years of practice	(ρ = 0.115; *p* = 0.088)
Attitudes/Years of practice	(ρ = −0.090; *p* = 0.182)

**Table 5:** Differences between variables.

	Knowledge		Differences	Attitudes			Differences
Demographics of *n* = 225 (*n*)	(%)	(M)		(%)	(M)		
Town:			t = 1.94 (df = 222) *p* = 0.054				t = 0.779 (df = 223) *p* = 0.437
Košice (149)	46.88	12.19		68.69	35.72		
Prešov (76)	43.58	11.33		66.17	34.41		
Position:			t = 1.356 (df = 216) *p* = 0.177				t = 0.984 (df = 22.38) *p* = 0.336
Nurse (198)	46.27	12.03		68.29	35.51		
Nurse- manager (21)	42.50	11.05		61.63	32.05		
Ward:			F = 2.07 (df = 7) *p* = 0.048 *				F = 1.55 (df = 7) *p* = 0.152
Surgical (55)	47.00	12.22		69.70	38.47		
Medical (32)	48.31	12.56		61.48	31.97		
Intensive (26)	44.54	11.58		64.50	36.12		
Geriatrics (16)	47.62	12.38		73.33	38.13		
Trauma (24)	49.35	12.83		57.21	32.04		
Neurology (29)	39.65	10.31		70.62	36.72		
Physiotherapy (10)	46.15	12.00		63.46	33.00		
Oncology (29)	42.58	11.07		59.48	33.31		
Education:			F = 2.51 (df = 5) *p* = 0.031 *				F = 0.06 (df = 5) *p* = 0.998
Sec. nursing school (39)	49.73	12.93		62.32	34.90		
Higher diploma (36)	43.38	11.28		67.67	35.19		
Specialty (57)	47.31	12.30		68.62	35.68		
Undergrad (BSc.) (52)	42.46	11.04		68.27	35.50		
Postgrad (MSc.) (36)	45.19	11.75		66.62	34.64		
Postgrad (DN) (4)	53.85	14.00		71.15	37.00		
Education in PU management:			F = 0.45 (df = 3) *p* = 0.718				F = 0.65 (df = 3) *p* = 0.858
General (at school) (166)	46.04	11.97		67.35	35.02		
Course (3)	47.42	12.33		80.77	42.00		
Continual education (46)	45.23	11.76		68.94	35.85		
Other (2)	36.54	9.50		82.69	43.00		

Notes: * *p*< 0.05.

## Data Availability

The data presented in this study are available on request from the corresponding author. The data are not publicly available due to General Data Protection Regulation (GDPR).
